# Sternal Aspiration of Bone Marrow in Dogs: A Practical Approach for Canine Leishmaniasis Diagnosis and Monitoring

**DOI:** 10.1155/2013/217314

**Published:** 2013-09-28

**Authors:** Rosa Paparcone, Eleonora Fiorentino, Silvia Cappiello, Manuela Gizzarelli, Luigi Gradoni, Gaetano Oliva, Valentina Foglia Manzillo

**Affiliations:** ^1^Department of Veterinary Clinical Science, Faculty of Veterinary Medicine, 80137 Naples, Italy; ^2^Unit of Vector-Borne Diseases & International Health, MIPI Department, 00161 Rome, Italy

## Abstract

Bone-marrow aspirate material is commonly considered as one of the most sensitive tissues for a reliable diagnosis of leishmaniasis. The procedure herein described may permit less experienced veterinarians to be familiar with a quick and safe assessment method for leishmaniasis diagnosis in their patients. Animals are positioned in right lateral recumbency, and the area corresponding to the second, third, or fourth sternebra is identified and aseptically prepared. A 18-gauge needle connected to a 10 mL syringe is driven through the skin, up to the bone wall, and firmly pushed forward while rotating. Entry into the sternebra's cavity is clearly perceived by the fall of resistance offered by the cortex. Some 2,500 sternal bone-marrow samplings were safely and efficiently performed on 887 dogs of different breeds and aging from 6 months to 14 years, during eight years of clinical activity for routine diagnosis of canine leishmaniasis in pets or for the efficacy evaluation of anti-*Leishmania* immunobiologicals in dogs naturally exposed to parasite transmission. Most of the samples (1716) were from 387 dogs enrolled for anti-*Leishmania* vaccine studies. The safety of the method was particularly assessed on these dogs that as per study protocol were submitted to repeated bone-marrow aspirations (2–4 per year) in follow-up examinations.

## 1. Introduction

Canine leishmaniasis (CanL) has emerged as a major veterinary and public health problem in endemic areas but also in nonendemic ones where individual clinical cases or outbreaks of disease are reported, such as in northern Europe, the USA, and Canada [1–3]. Diagnosis of leishmaniasis in dogs should be based on an integrated approach considering signalment, history, clinical findings, and results of laboratory analyses aiming at *Leishmania* detection and/or at the evaluation the host's immune responses [4]. Parasite detection can be achieved by microscopic demonstration of *Leishmania *amastigotes in macrophages of affected tissues. In dogs without clinical signs involving organs or tissues but yet suspected as having leishmaniasis because of exposure to infection risk or clinical recovery following drug treatment, a diagnostic sample should be obtained from tissues where parasites are more likely to be detected, such as spleen, bone marrow, lymph nodes, or buffy-coat from peripheral blood, in descending order of diagnostic sensitivity, respectively, [5, 6]. Part of the material sampled for cytology evaluation can be stored and, in case of negative microscopy, it can be sent to a reference laboratory for molecular diagnosis (e.g., a PCR assay for detection of leishmanial DNA). It should be pointed out that PCR assays on bone-marrow or lymph-node aspirates were shown by far more sensitive microscopic detection of *Leishmania* in smears or culture from these tissues [7]. However, lymphoid tissue material could be difficult to obtain in sufficient amount when palpable nodes are not enlarged. It should be noted that lymph-node enlargement is a clinical sign that may become evident only several months after infection [8]. The aim of our paper is first to describe in a short guideline format a technique for sternal bone-marrow aspiration proved to be safe and easy to perform; second, to provide validation results indicating the usefulness of the technique for parasitological examinations necessary to CanL diagnosis and monitoring in clinical practice.

## 2. Material and Methods

2,500 sternal bone-marrow samplings were performed on 887 dogs during eight years of clinical activity for routine diagnosis of canine leishmaniasis in pets or for the efficacy evaluation of anti-*Leishmania* immunobiologicals in dogs naturally exposed to parasite transmission. The dogs were aged 6 months-14 years and were of both sexes and of different breeds. Most of the samples (1716) were obtained from 387 dogs enrolled in five clinical trials approved by the Italian Ministry of Health and designed for testing immunobiologicals against CanL. Of them, 237 dogs were Beagles, whereas 150 belonged to 30 different breeds. The studies consisted of long-term follow-up evaluation (from 2 to 4 years) starting from a naive condition followed by natural exposure to infection transmission in endemic foci of CanL. Due to the longitudinal nature of these studies, dogs were submitted to frequent bone-marrow aspiration, for an average of 3 aspirations per year.

Owner dogs coming for clinical suspicion of CanL were usually submitted to a single bone-marrow aspiration, except for those that required a reevaluation of parasitological load during or after therapy. 

### 2.1. Technique for Sternal Bone-Marrow Aspiration

Equipment includes sterile gloves, a 18-gauge needle, a 10 mL syringe, and sterile gauze. No pharmacological sedation was usually required because of the quick procedure that may last 1-2 minutes. Slight sedation by medetomidine (Domitor; Pfizer) was only necessary in aggressive or agitated dogs. Pain control was obtained with butorphanol (Dolorex; Intervet) administered by intramuscular route (i.m.) 10–15 minutes before the sampling.

**Figure 1:**
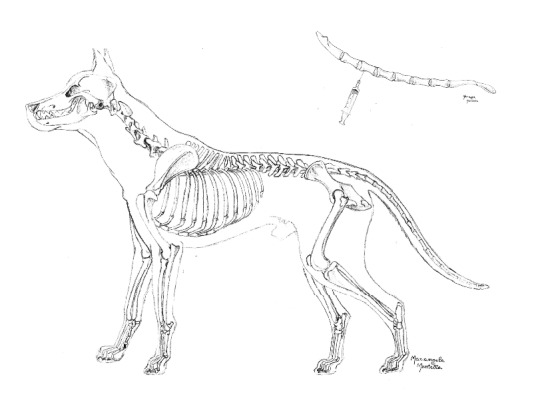
Anatomical site for sternal aspiration.

**Figure 2:**
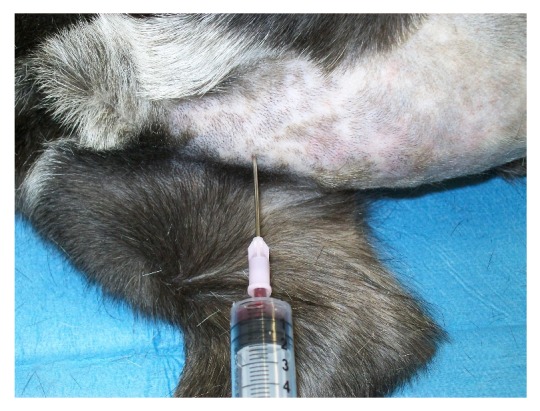
Bone marrow aspirate with the dog positioned in right lateral recumbency.

Briefly, the animals were positioned in right lateral recumbency with the right foreleg kept forward and the left foreleg kept backward, both parallel with the body axis. The area corresponding to the second, third, or fourth sternebra (Figure 1) was identified by palpation, clipped, and aseptically prepared. The needle was threaded through the skin up to the sternebra's wall and firmly pushed forward, while rotating (Figure 2). Entry into the bone cavity is clearly perceived thanks to the fall in resistance offered by the bone cortex. Approximately 5 mL of vacuum was then applied for 5 to 10 seconds depending on the volume of bone-marrow material required, usually around 0.5 mL. In some cases the aspiration of material may fail at the first attempt due to the incorrect insertion of the needle or to the needle obstruction by bone splinters. In these circumstances, it is sufficient to repeat quickly the procedure after changing the needle. This inconvenience occurred in about 5% of dogs.

A conventional Giemsa blood staining was then applied; in rare occasion a Diff-Quick staining was performed as alternative, with no difference of smears quality. Smears were first observed with low-power objective to gain an appreciation of the overall cellularity and determine the adequacy of megakaryocyte number. For each dog 3 appropriated slides were considered, and all of them were scanned using a high microscopic magnification (100x) to determine a complete 500 cell differential counts, the myeloid to erythroid ratio calculation, and the final interpretation of the cytologic findings. Parasitological examination was performed with the same magnification objective to show the presence of *Leishmania* amastigotes within macrophages or free in the extracellular environment, after the macrophages membrane destruction. Parasites count was done using the technique described by Chulay and Bryceson [9]. To avoid false-positive results a total number of 5 amastigotes per slide was considered as positive result. 

### 2.2. *Leishmania* Search in Bone-Marrow Material

In dogs enrolled in clinical trials of immunobiologicals against CanL, bone-marrow samples (usually more than 400 *μ*L) were obtained regardless of the clinical condition. Samples were used for *Leishmania *nested (n)-PCR examination as previously described [10]. As per protocol, bone-marrow samples were kept frozen at −35°C pending DNA extraction and PCR analysis.

In pets admitted to bone-marrow aspiration after owner's consent for parasitological investigations, parasitological diagnosis was performed by direct microscopy, following the staining routine procedures and microscopy observation previously reported. 

## 3. Results

By the end of the studies, 371/1716 bone-marrow n-PCR examinations were found positive for leishmanial DNA (21.6%), which included also several clinically healthy dogs in the early stages of infection. Except for a dog fraction that showed intermittent bone-marrow positivity during repeated investigations, n-PCR was constantly positive when a well-established infection was confirmed by a standard serological assay (immunofluorescent antibody test, IFAT cutoff 1 : 160) at titers higher than 1 : 160 [11]. Our PCR laboratory method excluded the possibility that “transient” n-PCR positives were false positives due to contamination. These finding may be explained by a degree of dog's resistance to primary infection, in which the parasite load in BM dropped to undetectable for a variable period of time until the number of organism increased again [11].

On the remaining 502 dogs (corresponding to 748 bone marrow samples) consisting of sick animals, *Leishmania *amastigotes were detected in 344/748 bone-marrow aspirate smears (46.0%), such rate being higher among dogs showing advanced stages of CanL and/or high IFAT titres. Also in these dogs concordance between IFAT titers higher than 1 : 160 and cytological parasite demonstration was equal to 100%, when anti-*Leishmania* antibodies were detected at first clinical examination in sick dogs.

No significant accidents due to the technical procedure were recorded, including those dogs submitted to more than 4 samplings per year. 

## 4. Conclusions

Diagnostic methods for diagnosis of CanL infection are grouped into 2 main categories: direct methods (cytological and histological microscopy evaluation, parasite culture, qualitative or quantitative PCR assays, and xenodiagnosis through the use of colonized phlebotomine sand flies) and indirect methods (different serological assays and *in vivo* or *in vitro* evaluation of cellular immune responses) [4]. Regarding direct methods, it is generally accepted that bone marrow and spleen are tissues with the highest density of *Leishmania* amastigotes. Lymph node tissue is similarly rich in parasites especially in sick dogs [6]; however, lymphoid material aspiration may prove difficult when lymph nodes are not enlarged, such as in early infection stages or in chronic asymptomatic CanL condition. We have experience of several veterinarians who hesitate to approach bone-marrow aspiration as a routine aid to diagnosis. Reasons for reluctance include lack of training and practice in sampling and in the microscopic evaluation of bone-marrow smear preparations, as well as fear for side effects or risky situations for the patients. On the other hand, main reasons for bone-marrow collection in diagnosis of CanL are several and can be summarized as follow: (1) to increase sensitivity of direct diagnostic methods, especially when sampling from palpable lymph nodes proves difficult, (2) to demonstrate parasite spread and multiplication during the early phases of infection, useful for CanL staging and prognostic for disease progression or drug failure, (3) to evaluate microscopically cytological changes usually associated with leishmaniasis, such as myeloid hyperplasiaor erythroid hypoplasia [12], thus, contributing to the disease diagnosis when parasites are not detected, and (4) to have an additional diagnostic tool for the evaluation of hematologic disorders such as nonregenerative anemia, thrombocytopenia, and neutropenia, which may occur in *Leishmania/Ehrlichia* coinfections commonly seen in endemic areas [13]. It has long been shown that differences in bone-marrow cellularity in samples collected from different sites are not significant [14, 15]. It follows that the preferable site to collect bone marrow depends mainly on technical aspects. Sites potentially available for bone-marrow sampling in dogs and cats include dorsal iliac crest, femoral shaft (via the intertrochanteric fossa), humeral shaft (via the lateral aspect of the greater tubercle), and costochondral junction (through the cartilage and into the rib marrow cavity). The iliac crest is considered the preferable site to collect bone marrow in medium- and large-sized dogs, while in small dogs and in cats, the trochanteric fossa of the femur or proximal humerus may be used [16, 17]. The sternal site is also indicated by some authors, but more often it is not listed among the preferred sites for collection, or it is considered dangerous for the risk of penetrating the thoracic cavity and injuring intrathoracic organs [17]. However, some clinicians have already shown that sternal aspiration could be considered easy to perform and effective for diagnostic purposes [14]. Although these authors have shown the feasibility of sternal sampling in dogs, they performed this technique on a limited number of animals, so that their results cannot be considered conclusive. Furthermore, in the protocol described by these authors, dogs were anesthetized and the bone-marrow aspiration was performed with the aid of an Illinois sternal needle. Both these practices make the method proposed less practical than the one proposed in our study. In fact, anesthesia is not always without risk, and an 18-gauge needle is more manageable and less traumatic than an Illinois sternal needle. Our study demonstrated on a large number of dogs that the bone marrow sternal aspiration gives the possibility to avoid anesthesia with particular advantage for both dog's health and economic reasons. As in regard to the risk of cardiac or pulmonary injures, in our experience conducted on 889 dogs submitted to some 2,500 bone marrow aspirations, we never encountered these problems despite our large canine population included breeds of all sizes. In addition, as described by other authors the sternum is advantageously suitable for aspiration because the bone is softer than other size, and it is associated with less pain than from the ileum [18]. Furthermore, it has been demonstrated that smears quality and particle number are similar for all sites [19]. In conclusion, the described sampling technique of sternal bone marrow can be considered safe and easy to perform, providing a good representative sample of tissue for both parasitological diagnosis of CanL and the evaluation of hematopoietic cellularity of the patient. This easy and high performance diagnostic technique for detection of* Leishmania* parasite may be applied for both during clinical practice and for long-term studies that required repeated bone marrow samplings.
